# Comparison of open and intracorporeal modified ureterosigmoidostomy (Mainz II) after laparoscopic radical cystectomy with bladder cancer

**DOI:** 10.1186/s12957-021-02148-y

**Published:** 2021-02-20

**Authors:** Duo Zheng, Junyao Liu, Gongjin Wu, Shujun Yang, Chuang Luo, Tianci Du, Yao Luo, Junsheng Bao, Junqiang Tian, Zhiping Wang, Panfeng Shang, Zhongjin Yue

**Affiliations:** Department of Urology, Institute of Urology, Key Laboratory of Urological Diseases of Gansu Province, Lanzhou University Second Hospital, No.82 Cui Ying Gate, Cheng guan District, Lanzhou, 730030 Gansu China

**Keywords:** Ureterosigmoidostomy, Urinary diversion, Mainz pouch II, Laparoscopy, Radical cystectomy, Urothelial carcinoma of the bladder

## Abstract

**Objective:**

To compare perioperative and oncologic outcomes of open modified ureterosigmoidostomy urinary diversion (OMUUD) and intracorporeal modified ureterosigmoidostomy urinary diversion (IMUUD) following laparoscopic radical cystectomy (LRC).

**Patients and methods:**

We retrospectively reviewed our single institutional collected database patients undergoing LRC from October 2011 to October 2019. The perioperative characteristics were compared between OMUUD and IMUUD, and overall survival (OS) and progression-free survival (PFS) were evaluated by the Kaplan-Meier method.

**Results:**

Overall, 84 patients were included. OMUUD and IMUUD were performed in 63 (75%) and 21 (25%) patients, respectively. IMUUD patients demonstrated shorter postoperative length of stay (16.24 ± 3.91 days vs. 18.98 ± 7.41 days, *P =* 0.033), similar operation time (498.57 ± 121.44 vs. 462.24 ± 99.71, *P* = 0.175), similar estimated blood loss [400 (200–475) ml vs. 400 (200–700) ml, *P* = 0.095], and similar overall complication rate within 30 days (19.05% vs. 25.40%, *P* = 0.848) and 90 days (23.81% vs. 17.46%, *P* = 0.748). Complete urinary control rate was 87.3% (55/63) in the OMUUD group. In IMUUD, the complete urinary control rate was 90.5% (19/21). There was no significant difference in OS (*χ*^2^ = 0.015, *P* = 0.901) and PFS (*χ*^2^ = 0.107, *P* = 0.743) between the two groups.

**Conclusion:**

IMUUD postoperative recovery is faster; other perioperative outcomes and oncology results are not significantly different with OMUUD. It is indicated that IMUUD can be utilized safely and effectively in the urinary diversion after LRC.

## Introduction

Bladder cancer is the ninth most common malignant tumor of the urinary system in the world [1]. Although the incidence of bladder cancer in China is lower than that in Euro-American countries, the morbidity of this disease is increasing year by year in recent years [2]. Radical cystectomy with pelvic lymph node dissection is supposed to be the gold standard of muscle-invasive bladder cancer and high-risk non-muscle-invasive bladder cancer [3]. The prevalence of laparoscopic radical cystectomy in the past three decades, compared with open radical cystectomy, has the advantages of less intraoperative blood loss and faster postoperative recovery [4].

In general, extracorporeal urinary diversion (ECUD) was operated after LRC in most medical centers because of its complex and time-consuming constructive procedure. Intracorporeal urinary diversion (ICUD) represents a viable alternative to ECUD and has the probable superiority in a smaller incision, relieving pain, reduced intestinal exposure, and reduced risk of body fluid out-of-balance [5]. In the majority of cases, the primary option for intracorporeal urinary diversion is the ileal conduit [6, 7] or orthotopic neobladder [8, 9].

The modified ureterosigmoidostomy (Mainz II) is a simple and reproductive operation technique, with good results in safety and feasibility [10]. As a capable alternative type of continent urinary diversion, it is the main mode of urinary diversion in our center. Our institution completed intracorporeal modified ureterosigmoidostomy urinary diversion (IMUUD) after LRC more than twenty cases since 2011. In this study, we will introduce our single-center experience with IMUUD and evaluate the safety and feasibility compared with open modified ureterosigmoidostomy urinary diversion (OMUUD).

## Patients and methods

From October 2011 to October 2019, 84 patients with bladder who received LRC with Mainz Pouch II were enrolled in this study, of whom 63 patients underwent LRC with OMUUD and 21 patients underwent LRC with IMUUD. The doctor told the patients the advantages and disadvantages of the two types of surgery, and then, the patient chooses the type of surgery. The premise is that the patient’s cardiopulmonary function can suffer from the surgery.

The following are the surgical indications: (a) muscle-invasive bladder cancer pT2-T4a, N0-Nx, and M0 and (b) T1G3 and recurrent non-muscle-invasive bladder cancer that could be uncontrolled after transurethral resection of bladder tumor and intravesical instillation.

The following are the surgical contraindications: (a) anal sphincter dysfunction, (b) after pelvic radiotherapy, (c) sigmoid diverticulum, (d) chronic diarrhea, (e) the previous sigmoid colon and rectal surgical history, (f) renal dysfunction, serum creatinine > 200 μmol/l. All patients were confirmed to have bladder urothelial tumors by imaging (enhanced computed tomography or magnetic resonance imaging) and pathology (pathology after TURBT or cystoscope biopsy). The Clavien-Dindo classification was used to assess postoperative 30-day and 90-day complications [11].

The following are the preoperative preparation: (1) 500 ml saline enema, an upright position retained for 1 h for anal sphincter function test; (2) colonoscopy ruled out colorectal disease; (3) oral polyethylene glycol electrolyte powder to prepare the intestinal tract, does not advocate mechanical enema. The following are the postoperative treatment: (1) it was unnecessary to indwell gastrointestinal decompression tube intraoperatively and postoperative. The liquid diet was started on the first postoperative day, and the general diet was given at 1 week postoperation; (2) after the operation, the transurethral abdominal drainage tube was removed according to the condition of drainage fluid; (3) the anus canal and bilateral ureteric Mono-J catheter were removed at the same time after 10–14 days.

### Urinary diversion

#### OMUUD

After laparoscopic radical cystectomy with standard pelvic lymph node dissection [12], remove the midline lower abdominal incision, about the length of 6~8 cm; 10~15-cm lengths of the selected sigmoid colon and rectum respectively were folded in inverted U shape, and then, the seromuscular layer was sutured and fixed. Following the outline, the bowel is opened antimesenterically and detubularized with electrocautery; 3/0 polygalactin suture was used to side-to-side anastomosis the inner wall of the sigmoid colon and rectum to form the posterior wall of the new reservoir outside the abdominal cavity. The ureter was crossed a vessel-free area of the mesentery into an intraperitoneal position and anastomosed with the posterior wall of the new reservoir. The length of 2~3-cm submucosal tunnel ureteric implantation facilitated an anti-reflux mechanism. Two 6F Mono-J ureteric stents were inserted into bilateral ureters and connected with the anal tube by mersilk suture in the urinary reservoir (the anal canal should be selected more than 26F with 3~4 side holes, and head-end of anal canal must be placed in the urinary reservoir). The seromuscular layer was interrupted sutured with 3/0 polygalactin suture to close the anterior wall of the pouch and establish a low-pressure and large-capacity urinary reservoir. The anal canal and ureteric stents were drawn out transanally and properly fixed to the skin.

#### IMUUD

**Fig. 1:**
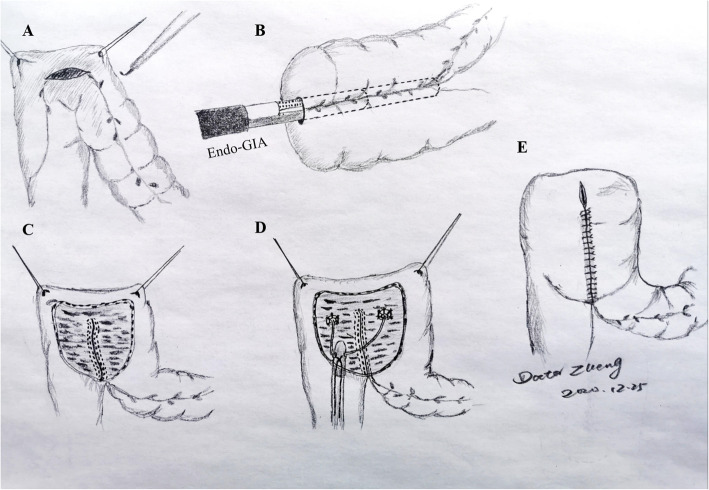
Schematic diagram of the surgical procedure

**Fig. 2:**
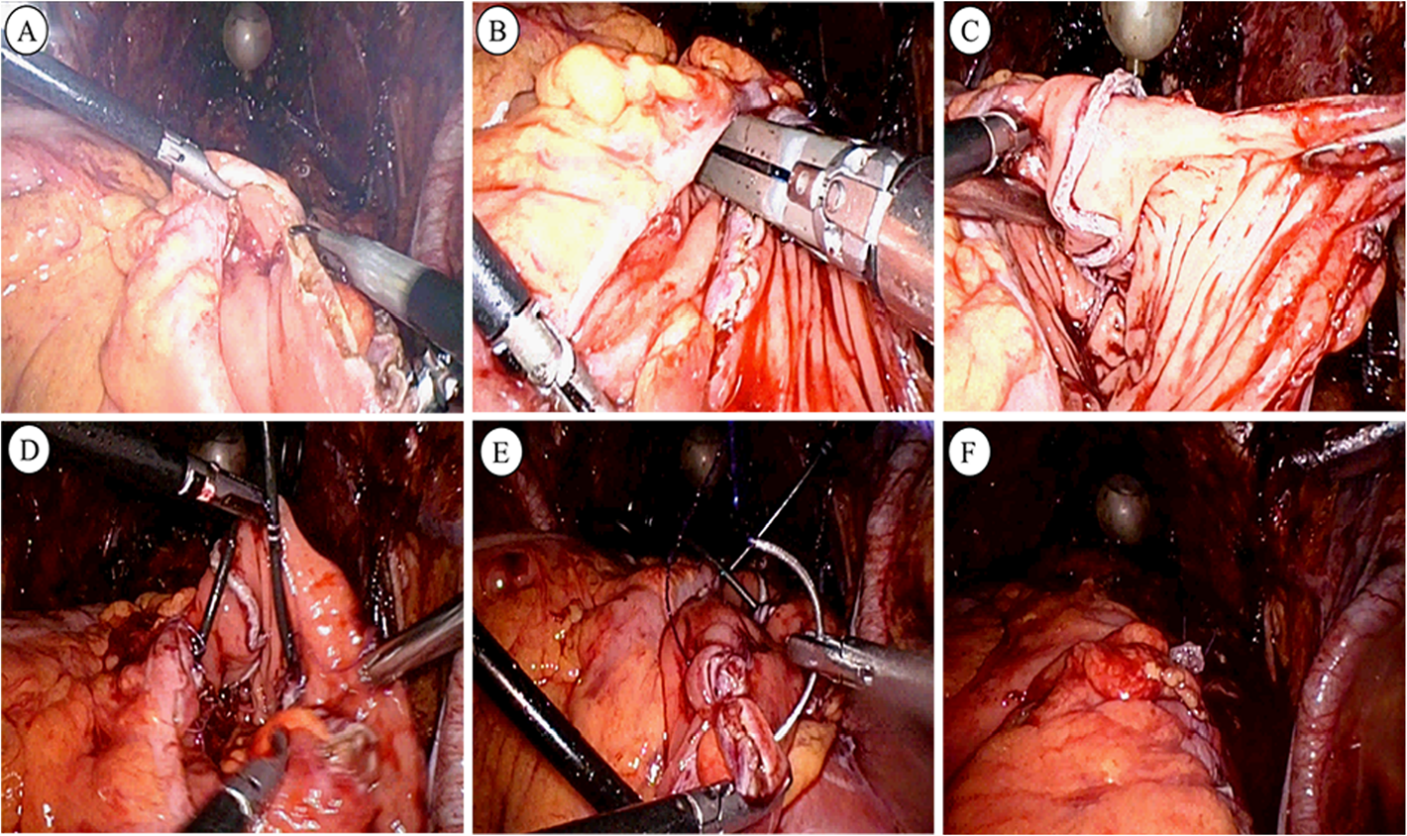
Pictures of the actual operation. **a** Following the outline, the bowel is opened antimesenterically and detubularized with electrocautery. **b**, **c** A new reservoir was made by Endo GIA stapler device. **d** Two 6F Mono-J ureteric stents were inserted into the bilateral ureters. **e** The seromuscular layer was interrupted sutured with 3/0 polygalactin suture to close the anterior wall of the pouch. **f** Operation completion status

After laparoscopic radical cystectomy with standard pelvic lymph node dissection, the selected sigmoid colon and rectum were folded into an inverted U shape. At the bottom of it, an incision about 4 cm along the colonic band was made. Two 60-mm laparoscopic Endo GIA stapler devices (Figs. 1, 2) were placed in the intestinal canal through the incision. The colonic band was put face to face into the cutting surface of the Endo GIA stapler device by adjusting the Endo GIA stapler device and the intestinal tube. After closed and cut, a urinary reservoir was quickly formed inside the abdominal cavity. The embedded-nipple ureteric implantation was applied to form an anti-reflux mechanism. The rest of the surgical procedure like OMUUD was completed in the abdominal cavity.

### Follow-up

All cases were followed every 6 months for the first 3 years, and then every year thereafter. Follow-up was carried out mainly through telephone followed by visits and outpatient reviews. The latest follow-up was conducted on February 29, 2020. OS is defined as the time from the start of the postoperative period to the onset of death; PFS is defined as the time from the start of the postoperative period to the onset of cancer progression or death.

Urinary continent is defined as not requiring the use of urinal pads on daily time.

### Statistical analysis

Continuous variables are presented as means (standard deviations (SD)) or median (interquartile range (IQR)), while categorical variables are expressed as absolute numbers and percentages. Pearson’s *χ*^2^ test or continuous correction *χ*^2^ test and independent sample *t* test or Mann-Whitney *U* test were used to evaluate categorical and continuous variables, respectively. Two-sided test *P* value < 0.05 was considered statistically significant. Statistical analyses were conducted using the SPSS software version 23.0 (IBM Corp, Chicago, IL, USA).

## Results

Overall, 84 patients were included. Of those, 63 (75%) and 21 (25%) patients were treated by OMUUD or IMUUD, respectively. There were no significant differences in baseline characteristics, according to age, gender, body mass index (BMI), American Society of Anesthesiologists Scores (ASA), and previous transurethral resection of bladder tumor (TURBT) (Table 1).

**Table 1:** Baseline characteristics of patients who received OMUUD or IMUUD

Items	OMUUD (*n* = 63)	IMUUD (*n* = 21)	*P* value
Age/years	61.71 ± 9.21	60.86 ± 10.07	0.719^a^
Gender, *n* (%)			1.000^b^
Male	50 (79.37)	17 (80.95)	
Female	13 (20.63)	4 (19.05)	
BMI (kg/m^2^)	22.56 ± 3.32	22.26 ± 2.63	0.708^a^
ASA score, *n* (%)			0.176 ^b^
I~II	55 (87.30)	15 (71.43)	
III	8 (12.70)	6 (28.57)	
Previous TURBT, *n* (%)			0.893^b^
No	43 (68.25)	14 (66.67)	
Yes	20 (31.75)	7 (33.33)	

^a^Independent sample *t* test

^b^Pearson’s *χ*^2^ test (or continuous correction *χ*^2^ test)

In terms of perioperative and pathological data, no difference was recorded for the pT stage, pN stage, lymph node yield, pathologic grade, and surgical margin (Table 2). Compared with OMUUD patients, IMUUD patients had similar operation time (498.57 ± 121.44 vs. 462.24 ± 99.71, *P* = 0.175), similar estimated blood loss [400 (200–475) ml vs. 400 (200–700) ml, *P* = 0.095], and shorter postoperative length of stay (16.24 ± 3.91 days vs. 18.98 ± 7.41 days, *P =* 0.033).

**Table 2:** The perioperative and pathological characteristics of patients who received OMUUD or IMUUD

Items	OMUUD (*n* = 63)	IMUUD (*n* = 21)	*P* value
pT stage, *n* (%)			0.709^a^
Ta /T1	13 (20.63)	4 (19.05)	
T2	30 (47.62)	12 (57.14)	
T3	16 (25.40)	3 (14.29)	
T4	4 (6.35)	2 (9.52)	
pN stage, *n* (%)			0.748^a^
Negative	52 (82.54)	16 (76.19)	
Positive	11 (17.46)	5 (23.81)	
Lymph node yield	9.44 ± 5.15	7.52 ± 3.92	0.122^b^
Pathologic grade, *n* (%)			0.109^a^
Low grade	18 (28.57)	10 (47.62)	
High grade	45 (71.43)	11 (52.38)	
Surgical margin, *n* (%)			1.000^a^
Negative	61 (96.83)	20 (95.24)	
Positive	2 (3.17)	1 (4.76)	
Time of operation	462.24 ± 99.71	498.57 ± 121.44	0.175^b^
Estimated blood loss	400 (200~700)	400 (200~475)	0.095^c^
Postoperative length of stay	18.98 ± 7.41	16.24 ± 3.91	**0.033**^**b**^
Perioperative transfusion, *n* (%)	25 (39.68)	4 (19.05)	0.085^a^

^a^Pearson’s *χ*^2^ test (or continuous correction *χ*^2^ test)

^b^Independent sample *t* test

^c^Mann-Whitney *U* test

All the observed morbidities were classified by the Clavien-Dindo classification for all patients in the two groups (Table 3). However, there were no significant differences between the two groups’ presented overall complications within 30 days of surgery (25.40% vs. 19.05%, *P* = 0.848) and 90-day complication (17.46% vs. 23.81%, *P* = 0.748).

**Table 3:** Postoperative outcome parameters

Items	OMUUD (*n* = 63)	IMUUD (*n* = 21)	*P* value
30-day complication rate, *n* (%)			
Clavien I	7 (11.11)	3 (14.29)	
Clavien II	8 (12.70)	1 (4.76)	
Clavien III	1 (1.59)	0	
Clavien IV	0	0	
Clavien V	0	0	
Overall complication rate, *n* (%)	16 (25.40)	4 (19.05)	0.848^a^
90-day complication rate, *n* (%)			
Clavien I	6 (9.52)	3 (14.29)	
Clavien II	0	0	
Clavien III	4 (6.35)	2 (9.52)	
Clavien IV	0	0	
Clavien V	1 (1.59)	0	
Overall Complication rate, *n* (%)	11 (17.46)	5 (23.81)	0.748^a^
Ureteric implantation site stricture, *n* (%)	4 (6.35)	2 (9.52)	1.000^a^

^a^Pearson’s *χ*^2^ test (or continuous correction χ^2^ test)

Urinary continence was available during daytime in 60 cases (95.2%) and occasional incontinence at night in 8 cases (12.7%), with a complete urinary control rate of 87.3% (55/63) in the OMUUD group. In the IMUUD group, daytime urinary continence rate was 100% and occasional incontinence at night in 2 cases (9.5%), with a complete urinary control rate of 90.5% (19/21).

**Fig. 3:**
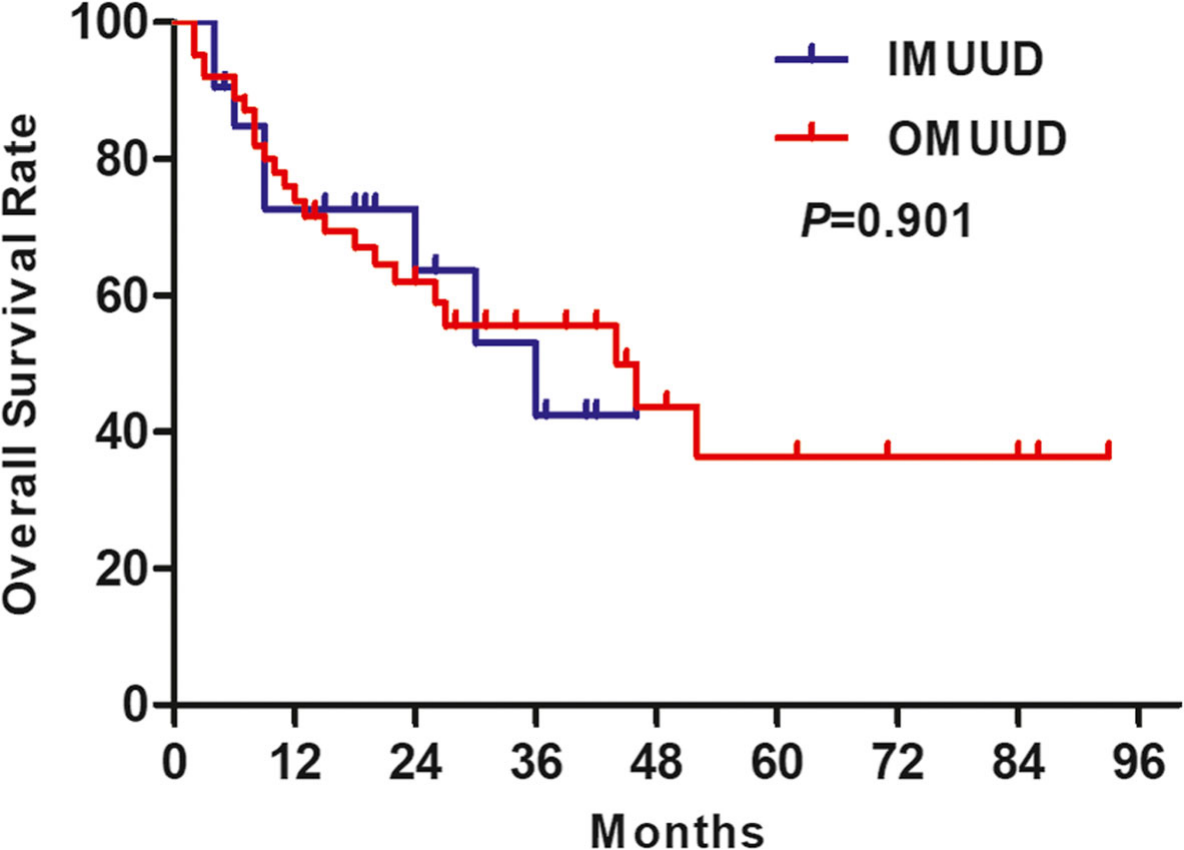
Kaplan-Meier curves of overall survival probability in patients who received OMUUD and IMUUD after LRC

**Fig. 4:**
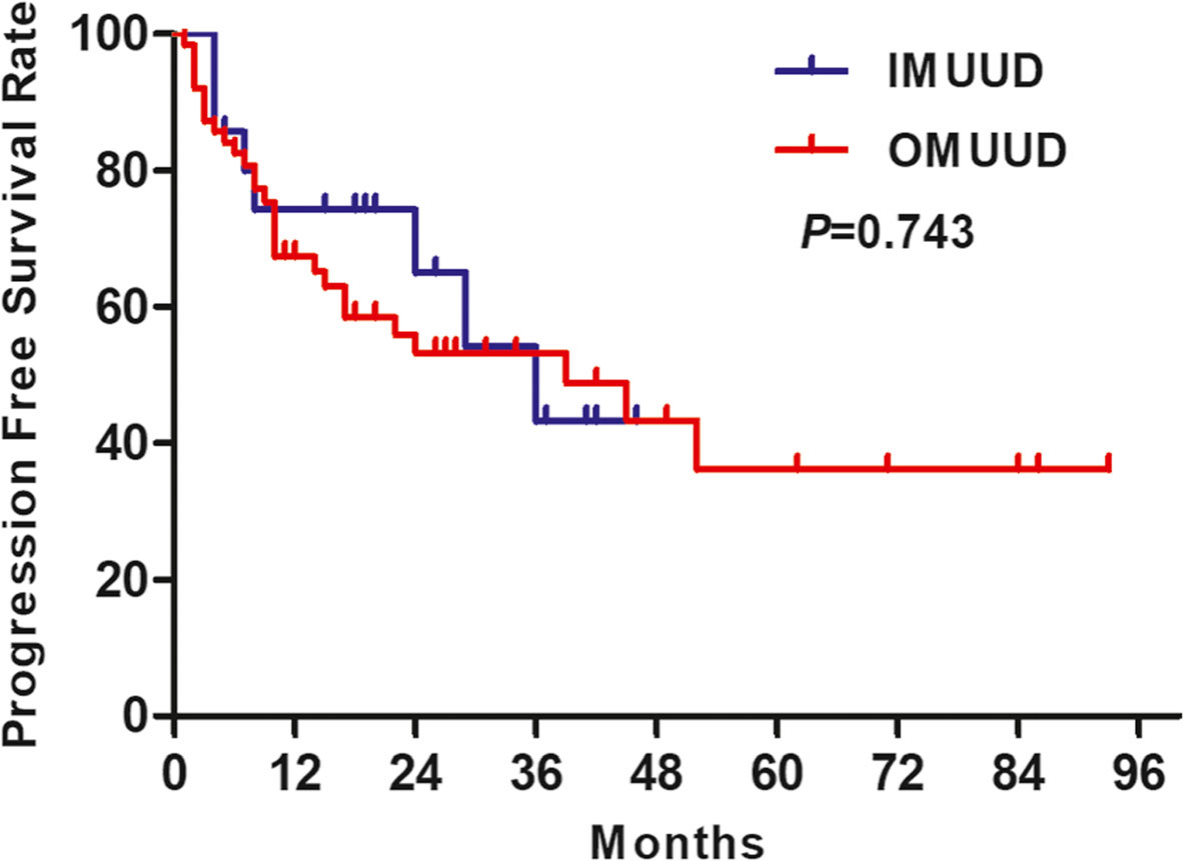
Kaplan-Meier curves of progression-free survival probability in patients who received OMUUD and IMUUD after LRC

The median follow-up time of this study was 15 months (interquartile ranges (IQR) 8~27.75 months). There were a total of 33 patient deaths and 36 patient disease progressions during the follow-up period. The 1-, 2-, and 3-year OS rates (Fig. 3) showed that there was no significant difference between the OMUUD group and IMUUD group (73.9% vs. 72.7%, 61.9% vs. 63.6%, 55.5% vs. 42.4%, log-rank test *χ*^2^ = 0.015, *P* = 0.901). The 1-, 2-, and 3-year PFS rates (Fig. 4) in the OMUUD and IMUUD groups were 67.4% vs. 74.3%, 53.3% vs. 65.0%, and 53.3% vs. 43.3%, respectively. The Kaplan-Meier curves for PFS showed no survival difference between the two groups (log-rank test *χ*^2^ = 0.107, *P* = 0.743).

## Discussion

Urinary diversion and bladder reconstruction after cystectomy are a subject that urologists have been studying for many years. Ureterosigmoidostomy, firstly reported by Simon [13] in 1852, was the first form of continent urinary diversion. Because of severe postoperative complications, such as reflux pyelonephritis, electrolyte imbalance, metabolic acidosis, impairment of renal function, renal calculus, and ureteric implantation site secondary tumor, the promotion of ureterosigmoidostomy was subject to limitations.

In 1993, Fisch et al. [14] carried out a modified ureterosigmoidostomy (Mainz II) based on the original ureterosigmoid anastomosis. The total length of 15–20 cm selected sigmoid colon and rectum was detubularized and side-to-side anastomosed to form a large-capacity and low-pressure reservoir without the need for colostomy, which did not disrupt the continuity of the intestinal tract [15]. This technique was a simple and elegant method to obtain satisfactory continence after operation [16]. Turk et al. [17] first described their experience of laparoscopic radical cystectomy with intracorporeal continent urinary diversion (rectal sigmoid pouch) in 5 patients. In their series, the results are promising, operation time was 6.9 to 7.9 h, and estimated blood loss was 190 to 300 ml.

In this study, the 30-day complication rate (23.81%, 20/84) was like other series of studies [10, 18, 19]. The most common early complications included wound infection, hypoproteinemia, fever, electrolyte disturbance, and intestinal obstruction. The 90-day complication rate was 19.05% (16/84). No difference in the complication rates at 30 days and 90 days was noted between the two groups. Especially, two patients developed metabolic acidosis during the follow-up in this study. They improved after the administration of oral sodium bicarbonate 600–2400 mg bid.

Ureteric implantation site stenosis as the most important late complication occurred in 6 patients (7.14%, 6/84). Is there a difference in anastomotic stenosis rate between different ureteric implantation techniques? Bastian et al. [20] found no significant difference in the incidence of anastomotic stenosis for three ureteric implantation techniques consist of Goodwin-Hohenfellner technique, Abol-Enein modification, and Le-Duc procedure. The anastomosis technique was enhanced with attention to the preservation of ureteral blood supply. The MAINZ II procedure ensures bowel continuity, and we can use the peristalsis of the upper colon to help the urinary reservoir to urinate. This will help to reduce urine reflux and inflammation of the anastomosis site thereby reducing the stenosis rate.

There is still controversy over whether to implement ECUD or ICUD. In most medical centers, ECUD is still the first choice of urologists, attribute to the advantage of shorter operation time [21]. Although the procedure of ICUD is time-consuming and laborious, the pressure provided by the pneumoperitoneum in laparoscopic surgery has a certain hemostatic effect, with advantages for better surgical vision, less intraoperative blood loss, and lower intraoperative blood transfusion rate. Besides, excessive bowel manipulation during the procedure and loss of body fluids contributed to the long postoperative bowel recovery time. In this study, the IMUUD group had a shorter postoperative length of stay (*P* = 0.033) compared to the OMUUD group. Although the difference in operative time between the two groups was not statistically significant (462.24 ± 99.71 vs. 498.57 ± 121.44, *P* = 0.175), it was a great labor intensity for the operator during the IMUUD. It is interesting that the operation times were not much longer for the IMUUD group. For this question, we propose the following hypotheses. First, our surgical operator completed cystectomy and LND faster for the IMUUD group because they did it after some learning curve. Second, the application of Endo GIA reduces the time of urinary diversion in the IMUUD group.

In order to reduce operation labor intensity, stainless steel staples have been generally utilized in the urinary tract after RC [22], and gradually, methods have emerged for applying linear cut closures to make a new reservoir. Gastrointestinal anastomosis (GIA) does not impact the time to bowl recovery following RC [23]. The use of the GIA stapler device was associated with a significant decrease in operation time and provides a good functional effect with acceptable complication rates [24]. Radical cystotomy is a relatively complex procedure with a long learning curve. The low-volume surgeons will benefit more by using stapling devices in radical cystectomy; it makes the surgical procedure safer and faster [25]. The application of GIA makes it easier for intracorporeal urinary diversion and reduces operator labor intensity. The nails of the Endo GIA stapler device will fall off by themselves in about 1 month, and there is no complication associated with reservoir stones in the follow-up.

Compared with other types of continent urinary diversion, low-pressure rectal reservoir represents an ideal choice for continent urinary diversion using an anal sphincter. Modified sigmoidorectal pouch can reduce the retrograde infection and renal function damage caused by urine reflux to the proximal colon. At the same time, it has a good urinary control rate, and the daytime urinary control rate can reach 100%. In these cases, urinary continence available during the daytime was 95.2% and 100%, with a complete urinary control rate of 87.3% (55/63) and 90.5% (19/21) between OMUUD and IMUUD, respectively.

This study, however, has some limitations. First, it was a retrospective controlled trial at a single institution with a small sample size. We can only collect the total operation time from the anesthesia record sheet, but not the time of urinary diversion. Second, despite our patient baseline and the pathological characteristics being similar between groups, there remains a degree of selection bias due to the non-randomized nature. In general, larger samples and multicenter randomized controlled trials are needed to further explore the effect evaluation and prognostic implications of patients with IMUUD.

## Conclusions

In summary, IMUUD postoperative recovery is faster, quality of life is higher, and oncology results are not significantly different. IMUUD may represent a viable alternative to open urinary diversion. Due to the small number of cases, the prognosis and associated complications remain to be further observed.

## Data Availability

Raw data may be requested from the authors with the permission of the institution.

## References

[1] Antoni S, Ferlay J, Soerjomataram I, Znaor A, Jemal A, Bray F (2017). Bladder cancer incidence and mortality: a global overview and recent trends. Eur Urol.

[2] Pang C, Guan Y, Li H, Chen W, Zhu G (2016). Urologic cancer in China. Jpn J Clin Oncol..

[3] Alfred WJ, Lebret T, Comperat EM, Cowan NC, De Santis M, Bruins HM, Hernandez V, Espinos EL, Dunn J, Rouanne M, Neuzillet Y, Veskimäe E, van der Heijden AG, Gakis G, Ribal MJ (2017). Updated 2016 EAU guidelines on muscle-invasive and metastatic bladder cancer. Eur Urol..

[4] Lin T, Fan X, Zhang C, Xu K, Liu H, Zhang J, Jiang C, Huang H, Han J, Yao Y, Xie W, Dong W, Bi L, Huang J (2014). A prospective randomised controlled trial of laparoscopic vs open radical cystectomy for bladder cancer: perioperative and oncologic outcomes with 5-year follow-upT Lin et al. Br J Cancer..

[5] Ahmed K, Khan SA, Hayn MH, Agarwal PK, Badani KK, Balbay MD, Castle EP, Dasgupta P, Ghavamian R, Guru KA, Hemal AK, Hollenbeck BK, Kibel AS, Menon M, Mottrie A, Nepple K, Pattaras JG, Peabody JO, Poulakis V, Pruthi RS, Redorta JP, Rha KH, Richstone L, Saar M, Scherr DS, Siemer S, Stoeckle M, Wallen EM, Weizer AZ, Wiklund P, Wilson T, Woods M, Khan MS (2014). Analysis of intracorporeal compared with extracorporeal urinary diversion after robot-assisted radical cystectomy: results from the International Robotic Cystectomy Consortium. Eur Urol.

[6] Kubota M, Kokubun H, Yamaguchi R, Murata S, Makita N, Suzuki I, Suzuki R, Abe Y, Tohi Y, Tsutsumi N, Sugino Y, Inoue K, Kawakita M (2020). Surgical outcomes and learning curve of totally intracorporeal ileal conduit urinary diversion following laparoscopic radical cystectomy at a single institution. Asian J Endosc Surg..

[7] Wang MS, He QB, Yang FY, Ping H, Xing NZ (2018). A retrospective study comparing surgical and early oncological outcomes between intracorporeal and extracorporeal ileal conduit after laparoscopic radical cystectomy from a single center. Chin Med J (Engl).

[8] Minervini A, Vanacore D, Vittori G, Milanesi M, Tuccio A, Siena G, Campi R, Mari A, Gavazzi A, Carini M (2018). Florence robotic intracorporeal neobladder (FloRIN): a new reconfiguration strategy developed following the IDEAL guidelines. BJU Int..

[9] Palleschi G, Pastore AL, Ripoli A, Silvestri L, Petrozza V, Carbone A (2015). Videourodynamic evaluation of intracorporeally reconstructed orthotopic U-shaped ileal neobladders. Urology..

[10] Hadzi-Djokic JB, Basic DT (2006). A modified sigma-rectum pouch (Mainz pouch II) technique: analysis of outcomes and complications on 220 patients. BJU Int..

[11] Clavien PA, Barkun J, de Oliveira ML, Vauthey JN, Dindo D, Schulick RD, de Santibañes E, Pekolj J, Slankamenac K, Bassi C, Graf R, Vonlanthen R, Padbury R, Cameron JL, Makuuchi M (2009). The Clavien-Dindo classification of surgical complications: five-year experience. Ann Surg..

[12] Bao J, Yue Z, Wu G, Shi W, Wang W (2017). Technique and results in total laparoscopic radical cystectomy with sigmoidorectal pouch (Mainz pouch II) - an initial experience. Exp Ther Med..

[13] Simon ST. Thomas's Hospital. Ectropia Vesicæ; (absence, of the anterior walls of the bladder and pubic abdominal parietes); operation for directing the orifices of the ureters into the rectum; temporary success; subsequent death; autopsy.Lancet. 1852,60:568-570. 10.1016/S0140-6736(02)63646-3.

[14] Fisch M, Wammack R, Müller SC, Hohenfellner R (1993). The Mainz Pouch II (sigma rectum pouch). J Urol..

[15] Fisch M, Hohenfellner R (2007). Sigma-rectum pouch (Mainz pouch II). BJU Int..

[16] Woodhouse CR, Christofides M (1998). Modified ureterosigmoidostomy (Mainz II)--technique and early results. Br J Urol..

[17] Türk I, Deger S, Winkelmann B, Schönberger B, Loening SA (2001). Laparoscopic radical cystectomy with continent urinary diversion (rectal sigmoid pouch) performed completely intracorporeally: the initial 5 cases. J Urol..

[18] Obek C, Kural AR, Ataus S, Coşkuner E, Demirkesen O, Citçi A, Onder AU, Solok V (2001). Complications of the Mainz pouch II (sigma rectum pouch). Eur Urol..

[19] Tyritzis SI, Hosseini A, Collins J, Nyberg T, Jonsson MN, Laurin O, Khazaeli D, Adding C, Schumacher M, Wiklund NP (2013). Oncologic, functional, and complications outcomes of robot-assisted radical cystectomy with totally intracorporeal neobladder diversion. Eur Urol..

[20] Bastian PJ, Albers P, Haferkamp A, Schumacher S, Muller SC (2004). Modified ureterosigmoidostomy (Mainz Pouch II) in different age groups and with different techniques of ureteric implantation. BJU Int..

[21] Patel HRH, Santos PB, de Oliveira MC, Müller S (2016). Is robotic-assisted radical cystectomy (RARC) with intracorporeal diversion becoming the new gold standard of care?. World J Urol..

[22] Kerbl K, Chandhoke P, McDougall E, Figenshau RS, Stone AM, Clayman RV (1993). Laparoscopic stapled bladder closure: laboratory and clinical experience. J Urol..

[23] Ghanaat M, Winer AG, Sjoberg DD, Poon BY, Kashan M, Tin AL, Sfakianos JP, Cha EK, Donahue TF, Dalbagni G, Herr HW, Bochner BH, Vickers AJ, Donat SM (2018). Comparison of postradical cystectomy ileus rates using GIA-80 versus GIA-60 intestinal stapler device. Urology..

[24] Muto G, Collura D, Simone G, Muto GL, Rosso R, Giacobbe A, Castelli E (2016). Stapled orthotopic ileal neobladder after radical cystectomy for bladder cancer: functional results and complications over a 20-year period. Eur J Surg Oncol..

[25] Tzortzis V, Gravas S, Mitsogiannis IC, Moutzouris G, Karatzas A, Leventis A, Mpouzalas I, Melekos MD (2008). Impact of stapling devices on radical cystectomy: comparative study between low- and high-volume surgeons. Urology..

